# Cooking Delicacy with Ice—Nanobubble Isolation Switches Stewing to ‘BBQ’

**DOI:** 10.3390/nano13030562

**Published:** 2023-01-30

**Authors:** Qiankang Si, Ruoyang Zhao, Feng Gao, Jun Guo, Feng Zhang, Liping Wang

**Affiliations:** 1 Wenzhou Institute, University of Chinese Academy of Sciences, Wenzhou 325001, China; 2 Key Laboratory of Optical Technology and Instrument for Medicine, Ministry of Education, University of Shanghai for Science and Technology, Shanghai 200093, China; 3 College of Life Science, University of Chinese Academy of Sciences, Beijing 101408, China

**Keywords:** freeze/thaw-induced cavitation, bulk nanobubbles, ice cooking, bubble isolation, stewing/barbeque switching

## Abstract

The key role of ice in cooking has been neglected. Here, we found negatively charged bulk nanobubbles (BNBs: average size ~60 nm and zeta potential <−20 mV) can be generated in ice-melted water through freeze/thaw-induced cavitation when we studied a local delicacy, ‘ice-stewed mutton’. Freeze/thaw-induced BNBs are so robust that they can, in turn, isolate food from water; in this way, they protect and enhance the delicacy by protecting protein structures and preventing flavorful components from being lost. In comparison to cooking with ordinary water, cooking with ice can switch ‘stewing’ to ‘BBQ’, which has been proved experimentally via diverse characterization from the nano to micro scale. This study not only provides a novel mechanism for ice-based cooking but also might shed light on the design of potential applications of BNBs in chemical engineering and biomedicine.

## 1. Introduction

Water is not only essential for life on Earth but also important for cooking food. Different forms of water have different roles in cooking. Ice-based food can be roughly classified into two categories: frozen drinks and ice-based desserts, which normally all contain ice as components. When using ice in chemical reactions, i.e., cooking, freeze/thaw-induced cavitation [1] will inevitably take effect. In this sense, gas bubbles are included in this topic.

In 1994, Parker et al. first proposed the concept of bulk nanobubbles (BNBs) [2], which has attracted much attention from many academic groups worldwide. BNBs usually refer to tiny bubbles with nanometer-sized diameters [3], which can be generated using a number of different physical and chemical methods. BNBs presented in water can exist stably for several hours [4,5]. Compared with traditional bubbles, BNBs not only have smaller diameters but also show more outstanding advantages in physicochemical properties [6,7], which means they have already been extensively studied [8] and have practical uses in many fields, such as in manipulating molecular self-assembly [9], therapeutic delivery [10,11,12], molecular imaging and diagnosis [13,14], and cancer therapy [15,16].

The study of BNBs requires interdisciplinary knowledge in both physics and chemistry, such as colloid and surface chemistry and soft matter physics. The tenderizing effect of BNBs has recently attracted attention in the food processing field [17], and BNBs can decrease thermal conductivity and have a certain thermal insulation effect [18,19], which exhibits a protective effect on meat tenderness and texture upon heating [20]. Here, we found a special food in the north of China in the Inner Mongolia autonomous region, named ice-stewed mutton, in which ice is used instead of water to cook mutton. We think this cooking method could be a typical example for us to use to begin our research.

People claim that mutton cooked in this way tastes better than with ordinary water. More interestingly, water is not lacking in these places, but this local delicacy can be traced back to a legend about Genghis Khan cooking around Lake Baikal, which is rich in ice (please refer to the Supporting Information). Aside from this historical myth, we are more interested in revealing the scientific secret behind the cooking method, since the study of cooking has already inspired many scientific discoveries such as Maillard reactions [21,22]. Inevitably, fresh mutton will become contracted when mixed with ice, and then, it will be swollen when heated, which has been considered by the local people as the main reason why it is a delicacy. However, this viewpoint cannot account for the tough-tasting–resistance phenomenon that occurs after meat is cooked for a long time; actually, ice-stewed mutton can still remain tender even after being boiled for a long time. We speculate that there must be some unnoticed differences between ice-melted water and ordinary water which play important roles in keeping the mutton tasting tender.

**Scheme 1:**
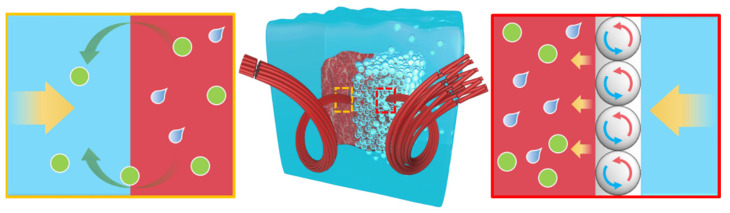
Schematic illustration of cooking with ice. When ice is used to cook instead of water, when the ice melts, air bubbles on the nanometer scale gather on the surface of food due to hydrophobic interaction. Interfacial BNBs can prevent nutrients from diffusing into water and protect protein structures, thus preserving more proteins and inorganic ions in the delicacy. Within typical meat, muscle fibers are arranged loosely, and the spaces can accommodate more juice; therefore, the delicacy tastes more tender.

We aimed to reveal how BNBs influence the ice-stewed mutton delicacy. We found BNBs formed an isolation layer between the food and water to protect the mutton via the following two aspects: One aspect was that BNB layers can effectively isolate water from mutton, thus avoiding direct heating transfer from water. Mediated by BNB, somehow, ‘stewing’ is switched to ‘barbeque’, which drastically slows down the rise in temperature and the protein denaturation in food. In this way, the shrinkage of the meat fibers also slows down, and the juice in the meat is retained to the maximum extent. Moreover, the BNB layers can significantly prevent substance exchange, thus preventing nutrients from dissolving into water; therefore, the flavorful contents will be preserved (Scheme 1).

## 2. Materials and Methods

### 2.1. Preparation of BNBs

BNBs were prepared by two ways: one was through a micro–nanobubble-generating device (called nanobubble water), and the other was through the freezing/thawing of ice (called ice-melting water). To prepare nanobubble water, the water pressure was increased to 0.8 MPa using the micro–nanobubble-generating device (NANO MF-5000, Shanghai Xingheng Technology Co., Ltd. Shanghai, China), the water temperature rose to about 60 °C, the flow rate of the water was kept at 5000 mL/min, and the self-priming height of the water in the machine reached 0.3 m. Then, the air and water were mixed using a gas–liquid mixing pump, and the air bubbles were reduced to smaller bubbles by the vortex collider of the micro-nanobubble-generating device. To prepare ice-melting water, pure water was firstly cooled to −20 °C to form ice cubes, and the ice cubes were used directly in the experiments.

### 2.2. Characterization of BNBs

Dynamic Light Scattering (DLS) and Zeta potential distribution measurements of samples were recorded using a ZEN3600 laser particle size analyzer (Malvern, UK). The hydrophobicity of the substrate surfaces fit the bubbles’ binding requirements. To mimic the surface of mutton, 1~2 mM short peptides GAV-9 with a sequence of VGGAVVAGV (purchased from Nanjing TGpeptide Biotechnology Co. Ltd, Nanjing, China) in 10 mM citrate buffer formed a self-assembled thin film on mica, altering the hydrophilic surface to a strongly hydrophobic surface. Then, ice-melting water was added to test the binding of BNBs. In situ 3-D topographic images were recorded on a commercial atomic force microscope (AFM, Nanoscope IIIa, Bruker, Billerica, MA, USA) equipped with a J-scanner and a liquid cell was employed for conducting the liquid phase tapping mode with silicon nitride cantilevers (0.58 N/m).

### 2.3. Mutton Cooking

The mutton samples were bought from supermarket (Baotou, Inner Mongolia, China). The visible membrane on the surface of meat samples and subcutaneous fat were removed, then the mutton was cut into pieces of (50 ± 2) g (n = 3). The test mutton was put in pure water, ice cubes, and nanobubble water, respectively, and the temperature was monitored with a K-type thermocouple probe digital thermometer. After the water (pure water, ice-melted water, and nanobubble water) was boiled, the meat samples were cooked for 5 min more and then were taken out. The water on the surface of the meat samples was dried with paper, and then, the cooked mutton was weighed and analyzed.

### 2.4. pH Measurement

A pH electrode was calibrated with a pH value of 4.0 and 6.8. After the cooked meat was taken out, the pH electrode was rinsed with distilled water and inserted into the meat sample; when the value of the pH meter was stable, the data were recorded. Three measurement points were selected for each meat sample.

### 2.5. Shear Force Measurement

The cooked meat samples were cut along the direction of the muscle fiber with the sample size: 5 × 1 × 1 cm. Then, the shear force was measured on the TA-XT2I physical property meter (TA.new plus, ISENSO. New York, NY, USA). The meat column was cut along the vertical direction of the muscle fiber with a muscle tenderness meter. Each meat sample was measured 5 times. Shear force measurement parameters: probe: HDP/BSW; speed before test: 2.00 mm/s; test speed: 2.00 mm/s; speed after test: 15.00 mm/s; pressing distance: 15 mm; load type: auto-20 g; data acquisition rate: 500 PPS.

### 2.6. Total Soluble Protein Content Measurement

A total of 0.5 g of the test meat sample was accurately weighed and put into a 100 mL centrifuge tube; then, 10 mL of ice bath potassium iodide extraction solution (1.1 M potassium iodide dissolved in 0.1 M potassium phosphate-buffered solution, pH 7.2) was added, and the test meat was homogenized. After centrifugation and extraction, the protein concentration in the supernatant was measured.

### 2.7. Myosin Content Measurement

A total of 1 g of the test meat sample was accurately weighed and put into a 100 mL centrifuge tube, and then, 10 mL of ice bath potassium phosphate-buffered solution (25 mM, pH 7.2) was added. The meat sample was homogenized twice for 30 s each time. The homogenate was placed on a shaking table and extracted for 12 h at 4 °C. The extracted solution was centrifuged (1500 r/min, 20 min), and the supernatant was separated. The protein concentration in the supernatant was determined using the biuret method.

### 2.8. SEM and EDS

The mutton was heated in BNBs and ordinary water, and the meat samples were taken out after being boiled for about 5 min. Then, the meat samples were fixed with 4% paraformaldehyde solution, followed by gradient dehydration with ethanol solution. After dehydration, the meat samples were sliced (longitudinal and cross-cut) and freeze-dried. The dried mutton samples were sprayed with gold and observed via scanning electron microscopy (SEM) (SU8010, HITACHI, Tokyo, Japan), and the element attribution was analyzed using energy-dispersive spectroscopy (EDS).

### 2.9. Histopathology

The cooked mutton samples were fixed with 4% paraformaldehyde solution and embedded in paraffin. They were sectioned into 5 μm thick slides. Dewaxing was performed in xylene. All samples were sequentially rehydrated with graded ethanol and water. Hematoxylin and eosin (H&E) staining (Shanghai Yisheng Biotechnology Co. Ltd. Shanghai, China) was then used to stain the samples, followed by being washed in water for 30 min. The slides were visualized using a light microscope (DP71, Olympus, Tokyo, Japan).

### 2.10. Statistical Analysis

All the experimental data in this study were analyzed using Student’s *t*-test. A * *p* < 0.05 was considered statistically significant. The results are expressed as mean ± standard deviation.

## 3. Results

### 3.1. Freeze/Thaw-Induced BNBs

**Figure 1:**
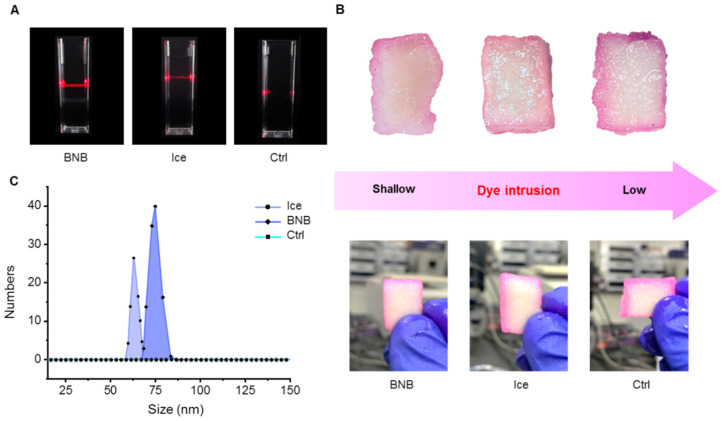
Characterization of freeze/thaw-induced BNBs. (**A**) Tyndall effect in different types of water; (**B**) dye intrusion to the fat cooked in different types of water; (**C**) the size distribution of nanobubble water and ice-melted water.

The Tyndall effect has been used to characterize nanobubbles [23,24]. To investigate the existence of BNBs in the ice-melted water, the Tyndall effect was tested in three types of water. A linear light route could be observed in ice-melted water and nanobubble water, while just two light spots were observed in ordinary pure water (Figure 1A). Meanwhile, we could see that the depth of dye penetration into the fat in ordinary pure water was the deepest, while the penetration depth was smaller in the boiled ice-melted water, and it was the smallest in the nanobubble water (Figure 1B). The images of the dye intrusion in fat revealed that the air BNBs gathered on the surface of the fat pieces, and the absorbed BNBs layer could act as a protective layer outside of mutton. Furthermore, the DLS measurements were used to determine the size distribution of BNBs; it could be seen that a single peak at around 60 nm appeared in ice-melted water, which was similar to that of the prepared nanobubble water (75 nm), while there was no peak observed in ordinary pure water (Figure 1C). The Tyndall effect results confirmed the existence of BNBs in ice-melted water. To observe the air bubbles in the system, the fat pieces were boiled in three types of rhodamine-B-dyed water (Figure S2); an interesting phenomenon was observed, as dense air bubbles appeared and absorbed on the surface of the fat pieces, except for in the ordinary pure water due to the hydrophobic effect. These results proved the presence of BNBs in ice-melted water.

**Figure 2:**
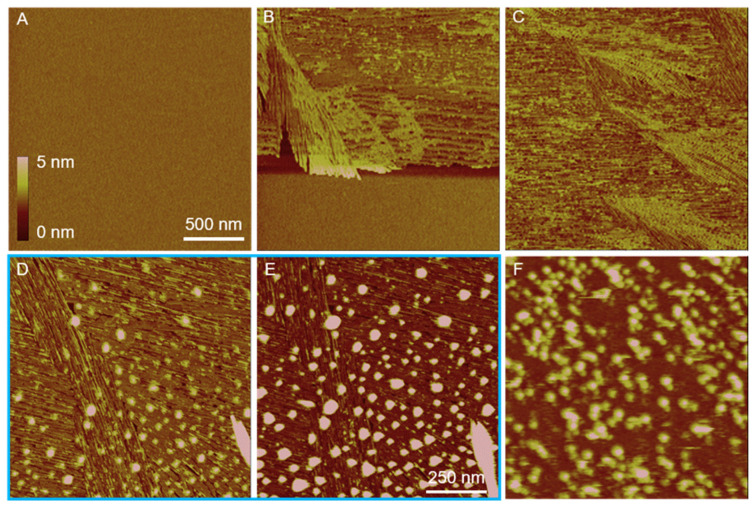
Interfacial BNB production and characterization via in situ AFM. AFM images of (**A**) bare mica; (**B**,**C**) short peptide GAV-9 self-assembled thin film on mica. (**D**,**E**) After the addition of ice-melting water, obvious nanobubbles rapidly absorbed onto the peptide self-assembled film. (**F**) After 12 min, the nanobubbles started to form an isolated layer between peptide film and bulk water until hard imaging via AFM. The height bar and scale bar are fit for all figures except (**D**,**E**), which are boxed in blue.

To observe clear 3D images of BNBs, the images of ice-melted water were tested using AFM. AFM was the first detection method used to discover and study interfacial nanobubbles, and AFM plays an important role in the study of basic properties of interfacial nanobubbles. Bare mica is a highly hydrophilic surface; nanobubbles from melted ice could not bind to it (Figure 2A), while short peptides (1 mM GAV-9 with a sequence of VGGAVVAGV in citrate buffer 10 mM) could form a self-assembled thin film on mica (Figure 2B,C), altering the hydrophilic surface to a strongly hydrophobic surface, which could mimic the surface of mutton. After the addition of ice-melted water, obvious nanobubbles rapidly absorbed onto the peptide film (Figure 2D,E); after about 12 min, the nanobubbles started to form an isolated layer between the peptide film and bulk water until hard imaging via AFM (Figure 2F), and obvious nanobubbles (around 30–80 nm) were observed.

### 3.2. Protective Effect of Interfacial BNBs through ‘BNB Isolation Wall’

Mutton is tender and rich in nutrition, including protein, carbohydrates, calcium, phosphorus, and inorganic salts. To explore the protective effect of air BNBs on mutton, EDS analysis was used to determine the element distribution in mutton. We could see that the nitrogen content in the mutton cooked in BNB water was 14.05%, which was much higher than that in the mutton cooked in ordinary pure water (10.65%) (Table 1). The nitrogen content can reflect the protein content in mutton; the higher nitrogen content indicated better protein preservation in mutton cooked in BNB water. The protein nutrients in mutton could be retained during the heating process due to the air BNB layers instead of being lost in the hot water. The absorbed BNB layers altered the interfacial transport phenomena and formed superhydrophobic surfaces. Meanwhile, the calcium, sodium, and chlorine contents in mutton cooked in BNB water were all much higher than those in the ordinary water condition. Sodium and chloride ions are both hydrated ions which can absorb polar water molecules, so higher contents of sodium and chloride ions could enhance the water retention capability of mutton. When the sodium and chloride ions entered the internal tissue, the osmotic pressure in the meat increased, and the water retention capability was improved accordingly, which improved the tenderness and taste of the meat. The calcium content in mutton cooked in BNB water was nearly twice that in ordinary pure water; a higher calcium content benefits to bones. The increased calcium could bind to myofibrils, which resulted in weakened and loose myofibril arrangements. The calcium ions could also activate calpain, and the meat protein was hydrolyzed, so the meat taste was more tender. The EDS results are very illustrative of the protective effect of nanobubbles; the protein and inorganic salt were well preserved in mutton rather than being diffused into water during the heating process, and the preserved inorganic salts were also beneficial to enhancing the taste and tenderness of the mutton.

**Figure 3:**
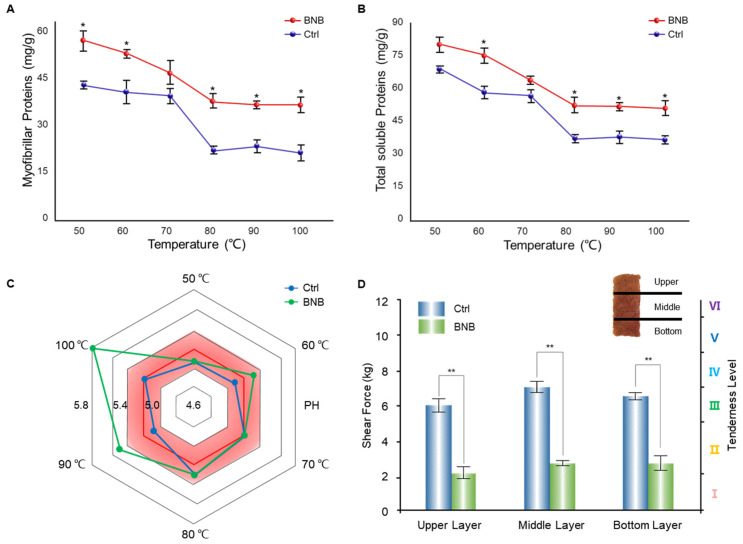
Effect of BNBs on food materials. (**A**) The myofibrillar protein and (**B**) the total soluble protein contents of mutton cooked in different types of water. (* significant difference *p* < 0.05). (**C**) The pH changes in mutton cooked in different types of water. In the anticlockwise direction, the heating temperature increased from 50 °C to boiling. The ring represents the pH value, which increases from inside to outside. (**D**) Shear force comparison of mutton cooked in different types of water. The mutton cooked in ordinary water and BNB water was cut along the vertical direction of the muscle, and in order to investigate it, three layers (top, middle, and low) were tested, and the corresponding tenderness level is shown in right coordinate. (** significant difference *p* < 0.01).

The spatial structure of proteins in mutton muscle plays a significant role in the tenderness of mutton, especially the content of myofibril proteins [25]. The increase in temperature will lead to the degeneration of myofibril proteins, and the degeneration and contraction of myofibril proteins will cause the heated mutton to taste hard and dry [26]. For the mutton cooked in nanobubble water, the myofibril and total soluble protein contents were much higher than those of mutton cooked in ordinary pure water (Figure 3A,B) during the whole heating process. The higher protein content might have been due to the protective effect of the BNB layer on the muscle surface, which could have slowed down the process of the degeneration and contraction of proteins. From the protein content profiles with the temperature, we could see that the myofibrillar protein and total protein contents decreased with the increase in temperature in both types of water. There was also a sharp decrease in ordinary pure water when the temperature increased from 70 to 80 °C, which might have been caused by the denaturation and contraction of myofibrils, while myofibrils decreased relatively slowly in the nanobubble water. The slower decrease in myofibrils illustrated that the BNB layer protected the protein in the mutton; the mutton was heated more evenly and the meat protein degenerated slower, which resulted in the mutton being more tender and tasting soft and juicy [27,28,29].

Tenderness is a key edible index to evaluate meat quality. The tenderness of meat refers to whether the meat is easy to cut, and the international standard unit is N/cm, which is expressed by shear force. Tenderness is usually evaluated by the difficulty in biting and chewing and the amount of residue after chewing. The shear force is the most commonly used index of meat tenderness. Generally, it is expressed as the maximum shear force required to cut off a meat section, and its unit is kg. There are six levels of tenderness: 0~2 kg is grade Ⅰ, which means extremely tender; 2~5 kg is grade Ⅱ, which means tender; 5~7 kg is grade Ⅲ, indicating average tenderness; 7~9 kg is grade Ⅳ, which means that it is a little rough and hard to bite; 9~11 kg is grade Ⅴ, which means it is rough and hard; >11 kg is grade Ⅵ, which means it is very hard to bite [30,31,32,33]. The shear force of cooked mutton was determined, and it could be seen that the average shear force of all three layers (top, middle, and low) were between 2 and 3 kg in nanobubble water, which was much smaller than that of mutton cooked in ordinary pure water (6~7 Kg, Figure 3D). These results indicated that the protective effect of BNBs on tenderness during the cooking process was quite obvious.

OH^-^ groups have the tendency to be absorbed on the interface of nanobubbles, which contribute to the negatively charged interface. From the Zeta potential values, we could see that the Zeta potential of ice-melted water was around −22 mV, which was similar to the Zeta potential of nanobubble water (−19 mV), while the ordinary pure water was neutral, and its Zeta potential was around 0 mV (Figure S1).

BNBs with negative interfaces absorbed on the surface of mutton might change the pH of the muscle and tune the taste. Many studies have shown that the final pH of muscle is closely related to the tenderness of meat. The effect of pH on muscle tenderness is mainly related to the change in static charge on the surface of the protein and is largely related to meat water retention. When the pH is around 5.2, the number of positive and negative charges carried by proteins in meat is nearly equal, and the mutual repulsion of the same charges become weak. The distance between protein molecules is shortened due to the weak repulsion, so the space for water molecules is reduced, and the water retention capacity is relatively low. As shown in the red area in Figure 3C, when the pH of meat was around this area, the meat tasted dry due to the low water retention. We could also see that the pH values of the mutton cooked in ordinary pure water were around 5.2, which were all in the red area, and the meat tasted dry. When the pH of the meat was below 7.0, the further away from 5.2 the pH was, the higher the water retention capability was, and the more tender and juicier the meat tasted [34]. The mutton cooked in nanobubble water showed a pH value higher than 5.2 at 50 °C (Figure 3B), which deviated much more from 5.2 than that of mutton cooked in ordinary pure water under the same conditions; the overall trends showed pH values all greater than 5.2, especially near the boiling temperature. The larger deviation from a pH of 5.2 in nanobubble water resulted in more water retention in the meat, reduced connective tissues, and improvements in the tenderness of meat.

### 3.3. Analysis of Meat Microstructures

**Figure 4:**
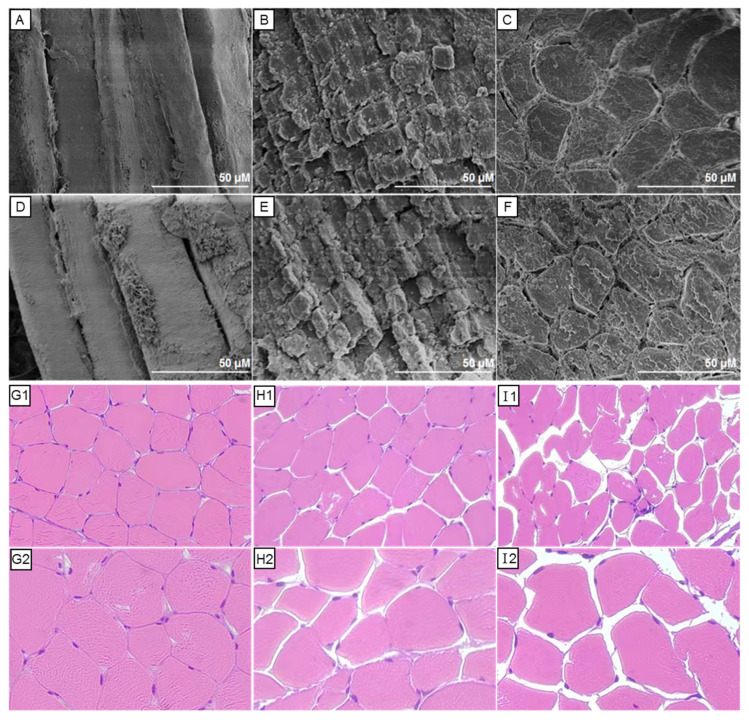
Characterization of muscles via SEM and H&E staining. (**A**,**B**) Longitudinal section and (**C**) cross-section analysis of mutton cooked in ordinary pure water; (**D**,**E**) longitudinal section and (**F**) cross-section analysis of mutton cooked in nanobubble water. The H&E staining images of mutton muscles cooked in (**G1**,**G2**) ordinary pure water, (**H1**,**H2**) ice-melted water, and (**I1**,**I2**) nanobubble water, respectively. The photos labeled with number 2 are zoomed in from those labeled with number 1.

To further explore the difference in the muscle structure and arrangement, SEM images of mutton cooked in different types of water were taken. When the cooked mutton muscle was magnified 1000 times, the microstructures of muscle fibers could be clearly observed. In the longitudinal section of mutton heated with ordinary pure water (Figure 4A), the muscle fiber bundles were arranged compactly, and the muscle fibers were in a tension state. Meanwhile, in the longitudinal sections of mutton heated in nanobubble water (Figure 4D), the arrangement of muscle fibers was not in a dense state, and obvious gaps could be observed between the muscle fiber bundles. When the mutton muscle fibers were magnified 10,000 times, the arrangement of the myofibrils could be revealed more clearly. The myofibrils arrangement was dense and compact in the ordinary pure water group (Figure 4B), the thick and tight muscle fibers showed great resistance to the teeth when chewed, and the taste of the mutton was hard and dry. Meanwhile, the myofibrils cooked in nanobubble water were not tightly arranged, and some spaces between the bright and dark bands could be observed (Figure 4E). The space between the fibers could accommodate more water, and when this mutton was chewed, the muscle fiber bundles were easier to bite, and the juice between the muscle fibers was squeezed out into the mouth [35], which meant chewing was easier and the mutton tasted much more tender.

SEM images of the cross-sections of mutton were also observed. Tightly packed myofibrils could be observed in the cross-sections of mutton in ordinary pure water. The muscle fibers were obviously smoother, and the arrangement of muscle fibers was compact and orderly (Figure 4C) compared with the mutton heated in nanobubble water (Figure 4F), and the juice in the meat was hardly retained in the ordered fibers. The severe protein denaturation resulted in the myofibrils shrinking violently, and the muscle fibers were arranged rigidly and tightly [28,36]. The spaces between the muscle fiber bundles cooked in nanobubble water were significantly bigger than those in mutton cooked in ordinary pure water. The larger gaps between muscle fibers cooked in nanobubble water could be observed clearly, and some holes also could be observed, which might have been caused by the effect of BNBs, which produced cavities when muscle fibers contracted. These bigger spaces and cavities could contain more water, and the water in the muscle had a kind of cooling effect due to the high specific heat capacity to water when the muscle proteins denatured during the heat process, which kept the protein denatured under relatively mild conditions, and the mutton tasted much more tender [36,37]. When the mutton was heated in nanobubble water, the protein denaturation and the contraction of mutton fibers slowed down, the increased spaces between the fibers could accommodate more juice, and the mutton tasted much more tender [38].

H&E staining images of the cooked mutton muscles were also determined. In the ordinary pure water cooked group, the muscles were orderly and tightly arranged, and nearly no gaps could be observed between the muscle fibers (Figure 4G). Meanwhile, in the ice-melted-water- and nanobubble-water-treated groups, obvious gaps could be observed between the muscle fibers. H&E staining showed that the gaps between the muscle fibers became bigger, and more voids appeared inside the muscle fibers, which was consistent with the SEM results (Figure 4H,I). The gaps between the muscle fibers were advantageous, as they held more juice to keep the mutton taste more tender.

## 4. Discussion

Freeze/thaw-induced BNBs are produced in a natural gas-liquid system. In ice-melted water, BNBs can be generated naturally, and BNBs play key roles in a local delicacy: ice-stewed mutton. The size distribution and Zeta potential both proved the existence of nanobubbles in the boiled ice-melted water, and the Tyndall effect could be observed in ice-melted water and nanobubble water. The BNBs tended to absorb on the mutton surface due to hydrophobic interaction, and the absorbed nanobubbles on mutton surface formed a protective interface between water and mutton. Under the protection of the absorbed BNB layer, the mutton could be heated uniformly, and the nutrition content in the mutton was inhibited from diffusing into the water. The proof of principle for the action of BNBs in ice-stewed mutton was strongly supported by our experiment results, as the experiment was deliberately designed for comparison with that cooked in ordinary water. First, both the myofibril and soluble protein contents were significantly higher. Second, the average shear force was two-fold smaller. Third, the muscle fiber bundles were significantly thinner and looser, and the gap between fiber bundles could accommodate more liquid. Owing to these differences, the ice-stewed mutton tasted tender and juicy even after being cooked for a long time. This work not only demonstrated the potential application of BNBs in both cooking and food processing fields but also revealed the mysterious properties of BNBs, which helps us gain a better understanding and control of BNBs. It can be predicted that BNBs will be effectively applied in sewage treatment, green cleaning, agriculture, medical health, and other fields.

## Figures and Tables

**Table 1:** The element distribution in food materials cooked in different ways.

Element	Concentration (wt. %)	
	BNB	Ctrl
N	14.05	10.65
Na	34.97	24.12
Ca	0.77	0.33
Cl	32.07	22.13

## Data Availability

The data presented in this study are available on request from the corresponding author.

## Supplementary Materials

The following supporting information can be downloaded at: https://www.mdpi.com/article/10.3390/nano13030562/s1, A context for “The legend of ice-stewed mutton”; Figure S1: Zeta potential distribution of different types of water; Figure S2: Evidence for BNBs adsorbed on food surfaces; Figure S3: The size distribution of nanobubbles under different conditions.

## References

[1] Sevanto S., Holbrook N.M., Ball M.C. (2012). Freeze/Thaw-induced embolism: Probability of critical bubble formation depends on speed of ice formation. Front. Plant Sci..

[2] Parker J.L., Claesson P.M., Attard P. (1994). Bubbles, cavities, and the long-ranged attraction between hydrophobic surfaces. J. Phys. Chem..

[3] Temesgen T., Bui T.T., Han M., Kim T.-I., Park H. (2017). Micro and nanobubble technologies as a new horizon for water-treatment techniques: A review. Adv. Colloid Interface Sci..

[4] Borkent B.M., Dammer S.M., Schönherr H., Vancso G.J., Lohse D. (2007). Superstability of surface nanobubbles. Phys. Rev. Lett..

[5] Seddon J.R., Kooij E.S., Poelsema B., Zandvliet H.J.W., Lohse D. (2011). Surface bubble nucleation stability. Phys. Rev. Lett..

[6] Sun Y.J., Xie G.Y., Peng Y.L., Xia W., Sha J. (2016). Stability theories of nanobubbles at solid-liquid interface: A review. Colloids Surf. A Physicochem. Eng. Asp..

[7] Li H., Hu L., Xia Z. (2013). Impact of groundwater salinity on bioremediation enhanced by micro-nano bubbles. Materials.

[8] Zhou L., Wang X., Shin H.-J., Wang J., Tai R., Zhang X., Fang H., Xiao W., Wang L., Wang C. (2020). Ultrahigh Density of Gas Molecules Confined in Surface Nanobubbles in Ambient Water. J. Am. Chem. Soc..

[9] Wang Y., Shen Z., Guo Z., Hu J., Zhang Y. (2018). Effects of nanobubbles on peptide self-assembly. Nanoscale.

[10] Xie X., Yang Y., Lin W., Liu H., Liu H., Yang Y., Chen Y., Fu X., Deng J. (2015). Cell-penetrating peptide-siRNA conjugate loaded YSA-modified nanobubbles for ultrasound triggered siRNA delivery. Colloids Surf. B Biointerfaces.

[11] Hernandez C., Gulati S., Fioravanti G., Stewart P.L., Exner A.A. (2017). Cryo-EM Visualization of Lipid and Polymer-Stabilized Perfluorocarbon Gas Nanobubbles—A Step Towards Nanobubble Mediated Drug Delivery. Sci. Rep..

[12] Cavalli R., Soster M., Argenziano M. (2016). Nanobubbles: A promising efficienft tool for therapeutic delivery. Ther. Deliv..

[13] Gao Y., Hernandez C., Yuan H.-X., Lilly J., Kota P., Zhou H., Wu H., Exner A.A. (2017). Ultrasound molecular imaging of ovarian cancer with CA-125 targeted nanobubble contrast agents. Nanomed. Nanotechnol. Biol. Med..

[14] Bosca F., Bielecki P.A., Exner A.A., Barge A. (2018). Porphyrin-Loaded Pluronic Nanobubbles: A New US-Activated Agent for Future Theranostic Applications. Bioconjug. Chem..

[15] Yin T., Wang P., Li J., Zheng R., Zheng B., Cheng D., Li R., Lai J., Shuai X. (2013). Ultrasound-sensitive siRNA-loaded nanobubbles formed by hetero-assembly of polymeric micelles and liposomes and their therapeutic effect in gliomas. Biomaterials.

[16] Jing H., Cheng W., Li S., Wu B., Leng X., Xu S., Tian J. (2016). Novel cell-penetrating peptide-loaded nanobubbles synergized with ultrasound irradiation enhance EGFR siRNA delivery for triple negative Breast cancer therapy. Colloids Surf. B Biointerfaces.

[17] Bhat Z.F., Morton J.D., Mason S.L., Bekhit A.E.D.A. (2018). Applied and emerging methods for meat tenderization: A comparative perspective. Compr. Rev. Food Sci. Food Saf..

[18] Wang X., Wang X., Muhoza B., Feng T., Xia S., Zhang X. (2020). Microwave combined with conduction heating effects on the tenderness, water distribution, and microstructure of pork belly. Innov. Food Sci. Emerg. Technol..

[19] Thomas O.C., Cavicchi R.E., Tarlov M.J. (2003). Effect of surface wettability on fast transient microboiling behavior. Langmuir.

[20] Maheshwari S., van der Hoef M., Prosperetti A., Lohse D. (2018). Dynamics of formation of a vapor nanobubble around a heated nanoparticle. J. Phys. Chem. C.

[21] Humphries C. (2012). Cooking: Delicious science. Nature.

[22] Barham P., Skibsted L.H., Bredie W.L., Bom Frøst M., Møller P., Risbo J., Snitkjær P., Mortensen L.M. (2010). Molecular gastronomy: A new emerging scientific discipline. Chem. Rev..

[23] Dhungana P., Bhandari B. (2021). Development of a continuous membrane nanobubble generation method applicable in liquid food processing. Int. J. Food Sci. Technol..

[24] Li T., Cui Z., Sun J., Jiang C., Li G. (2021). Generation of Bulk Nanobubbles by Self-Developed Venturi-Type Circulation Hydrodynamic Cavitation Device. Langmuir.

[25] Marino R., Albenzio M., Della Malva A., Santillo A., Loizzo P., Sevi A. (2013). Proteolytic pattern of myofibrillar protein and meat tenderness as affected by breed and aging time. Meat Sci..

[26] Ježek F., Kameník J., Macharáčková B., Bogdanovičová K., Bednář J. (2019). Cooking of meat: Effect on texture, cooking loss and microbiological quality—A review. Acta Vet. Brno.

[27] Weston A., Rogers R., Althen T. (2002). The role of collagen in meat tenderness. Prof. Anim. Sci..

[28] Malheiros J.M., Braga C.P., Grove R.A., Ribeiro F.A., Calkins C.R., Adamec J., Chardulo L.A.L. (2019). Influence of oxidative damage to proteins on meat tenderness using a proteomics approach. Meat Sci..

[29] Bertola N.C., Bevilacqua A.E., Zaritzky N.E. (1994). Heat treatment effect on texture changes and thermal denaturation of proteins in beef muscle. J. Food Process. Preserv..

[30] Wheeler T., Shackelford S., Koohmaraie M. (2001). Shear Force Procedures for Meat Tenderness Measurement.

[31] Warner R., Miller R., Ha M., Wheeler T.L., Dunshea F., Li X., Vaskoska R., Purslow P., Wheeler T. (2021). Meat tenderness: Underlying mechanisms, instrumental measurement, and sensory assessment. Meat Muscle Biol..

[32] Laville E., Sayd T., Terlouw C., Chambon C., Damon M., Larzul C., Leroy P., Glenisson J., Cherel P. (2007). Comparison of sarcoplasmic proteomes between two groups of pig muscles selected for shear force of cooked meat. J. Agric. Food Chem..

[33] Hopkins D., Hegarty R., Walker P., Pethick D.W. (2006). Relationship between animal age, intramuscular fat, cooking loss, pH, shear force and eating quality of aged meat from sheep. Aust. J. Exp. Agric..

[34] Thompson J. (2002). Managing meat tenderness. Meat Sci..

[35] Ertbjerg P., Puolanne E. (2017). Muscle structure, sarcomere length and influences on meat quality: A review. Meat Sci..

[36] Picard B., Gagaoua M. (2020). Muscle Fiber Properties in Cattle and Their Relationships with Meat Qualities: An Overview. J. Agric. Food Chem..

[37] Koohmaraie M., Kent M.P., Shackelford S.D., Veiseth E., Wheeler T.L. (2002). Meat tenderness and muscle growth: Is there any relationship?. Meat Sci..

[38] D’Alessandro A., Zolla L. (2013). Foodomics to investigate meat tenderness. TrAC Trends Anal. Chem..

