# Supplementary material for: Cooking Delicacy with Ice—Nanobubble Isolation Switches Stewing to ‘BBQ’
Source: Nanomaterials (Basel). 2023 Jan 30;13(3):562. doi: 10.3390/nano13030562 (PMC9920162; doi:10.3390/nano13030562)
Supplement: Supplementary file 1 [file nanomaterials-13-00562-s001.zip › nanomaterials-2159476-supplementary.pdf]

nanomaterials MDPI

Supplementary Materials

Cooking Delicacy with Ice—Nanobubble Isolation Switches Stewing to 'BBQ'

Qiankang Si [1,2,#], Ruoyang Zhao [1,3,#], Feng Gao [1], Jun Guo [1], Feng Zhang [1,2,*] and Liping Wang [1,*]

1 Wenzhou Institute, University of Chinese Academy of Sciences, Wenzhou 325001, China; siqiankang@gmail.com (Q.S.); zhaoruoyang@hotmail.com (R.Z.); 15849281587@163.com (F.G.); guojun-nbm@hotmail.com (J.G.)
2 Key Laboratory of Optical Technology and Instrument for Medicine, Ministry of Education, University of Shanghai for Science and Technology, Shanghai 200093, China
3 College of Life Science, University of Chinese Academy of Sciences, Beijing 101408, China
* Correspondence: fzhang@usst.edu.cn (F.Z.); lpwang-wiucas@ucas.ac.cn (L.W.)
# These authors contributed equally to this work.

The legend of ice-stewed mutton

According to the legend, Genghis khan went on an expedition around Lake Baikal area, and they did not get the town for a long time. The conditions were very tough, freezing cold, almost no food, and there was no water supply. Fire and ice are the only things they could get. The soldiers were very cold and hungry, Genghis khan ordered the soldiers to smash Lake Baikal ice, and put the ice and mutton in the bronze helmet. Then made a fire, the mutton is cooked when the ice water boiling. It was found that the mutton cooked in this way tasted not quite the same as usual, which was more delicious, fresh and tender. The mutton cooked in this way was slowly passed down by later generations, and which was very popular in north of China like Inner Mongolia autonomous region, named ice-stewed mutton.

Supplementary Figures

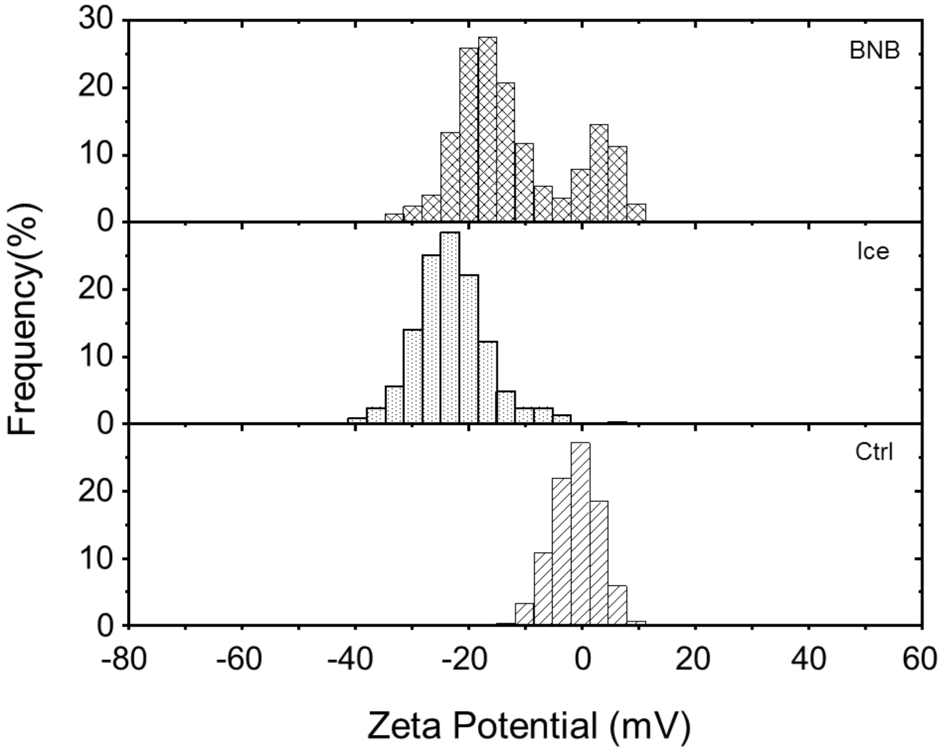

Figure S1. Zeta potential distribution of different types of water.

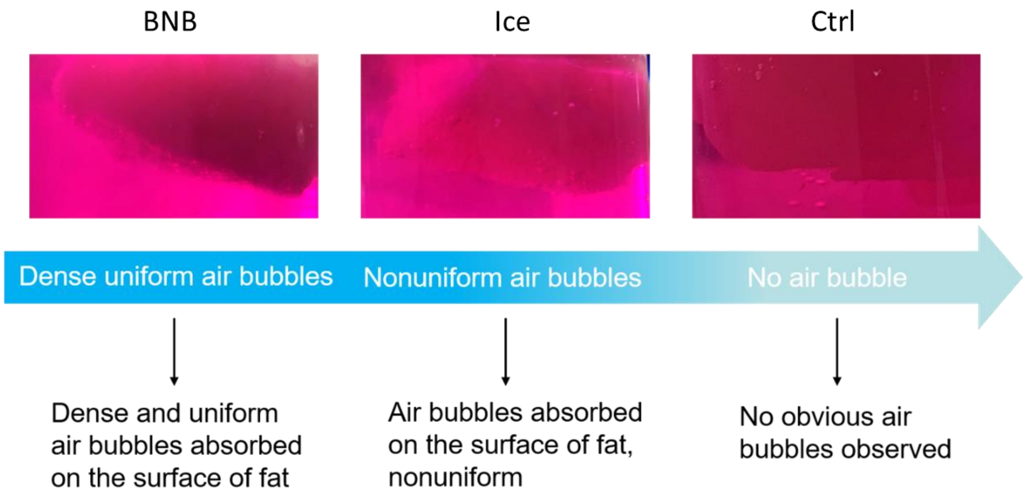

Figure S2. Evidence for BNBs adsorbed on food surface. Fat pieces were boiled in three types of rhodamine B dyed water, and the images were taken to observe the absorbed air bubbles on the surface of the fat pieces.

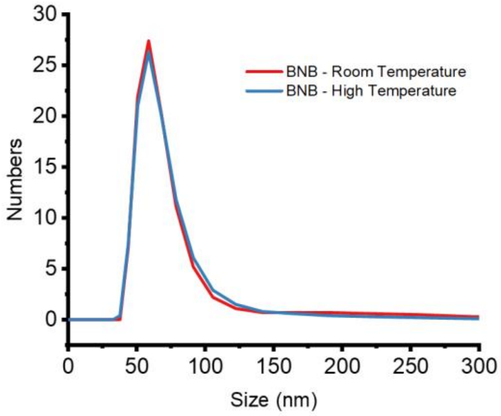

Figure S3. The size distribution of nanobubbles under different condition.
